# Tertiary syphilis in the lumbar spine: a case report

**DOI:** 10.1186/s12879-017-2620-5

**Published:** 2017-07-24

**Authors:** Yang Bai, Feng Niu, Lidi Liu, Hui Sha, Yimei Wang, Song Zhao

**Affiliations:** 1 Department of Cardiac Surgery, The First Hospital of Jilin University, Changchun, 130021 China; 2 Department of Spine Surgery, The First Hospital of Jilin University, Changchun, 130021 China; 3 Department of Pathology, The First Hospital of Jilin University, Changchun, 130021 China

**Keywords:** Syphilis, Lumbar, Spine, Charcot’s arthropathy

## Abstract

**Background:**

The incidence of tertiary syphilis involvement in the spinal column with destructive bone lesions is very rare. It is difficult to establish the correct diagnosis from radiographs and histological examination alone. Limited data are available on surgical treatment to tertiary syphilitic spinal lesions. In this article, we report a case of tertiary syphilis in the lumbar spine with osteolytic lesions causing cauda equina compression.

**Case presentation:**

A 44-year-old man who suffered with low back pain for 6 months and progressive radiating pain at lower extremity for 1 week. Radiologic findings showed osteolytic lesion and new bone formation in the parts of the bodies of L4 and L5. Serum treponema pallidum hemagglutination (TPHA) test was positive. A surgery of posterior debridement, interbody and posterolateral allograft bone fusion with instrumentation from L3 to S1 was performed. The low back pain and numbness abated after operation. But the follow-up radiographs showed absorption of the bone grafts and failure of instrumentation. A Charcot’s arthropathy was formed between L4 and L5.

**Conclusion:**

It is challenging to diagnose the tertiary syphilis in the spine. Surgery is a reasonable auxiliary method to antibiotic therapy for patients who suffered with neuropathy. Charcot’s arthropathy should be considered as an operative complication.

## Background

Syphilis is a sexually transmitted infection disease which caused by the spirochete Treponema pallidum. The World Health Organization (WHO) estimated that 5.6 million new cases of syphilis occurred among adolescents and adults worldwide in 2012 [1]. Though the incidence of primary and secondary syphilis is still increasing these years, the incidence of tertiary syphilis had remarkable decreased due to widespread availability of effective treatment. In this report, we present a case of tertiary syphilis in the lumbar vertebra.

## Case presentation

A 44-year-old man was hospitalized because of low back pain for 6 months and progressive radiating pain at lower extremity for 1 week. He had numbness below the knees, with two ankle progressively weakening and inability to walk. There was no fever, chills, cough, dyspnea, nausea and vomiting in the course of the disease. He had suffered “arthritis” in the right ankle for 6 years. Glans penis lesions were found about 8 years ago but self-healed. He has no history of tuberculosis. His vital signs were normal. The physical signs were severe limitation of all spinal movements in the lumbar region; pain on percussion over the lumbosacral area, which also produced radicular pain to lower extremity; there was partial sensory loss in the feet and distal parts of the legs; the ankle tendon reflexes were absent; the left ankle dorsiflexors power was weak; the right ankle was fixed in 90° of flexion. The straight leg raising test was bilaterally positive. The patient’s pupils, cardiovascular, pulmonary and abdominal examination were normal.

Laboratory examination showed that the white blood count, erythrocyte sedimentation rate, and C-reactive protein were normal. Serum treponema pallidum hemagglutination (TPHA) test was positive. The quantitative rapid plasma reagin (RPR) reaction of serum was positive (1:16). Cerebrospinal fluid (CSF) pressure was not elevated during lumbar puncture. The CSF had an increased protein level of 0.93 g/L, normal WBCs (5 × 10^6^/L) and normal glucose level (4.04 mmol/L). The RPR reaction of CSF was positive (1:2). The serum brucella agglutination test was negative.

**Fig. 1:**
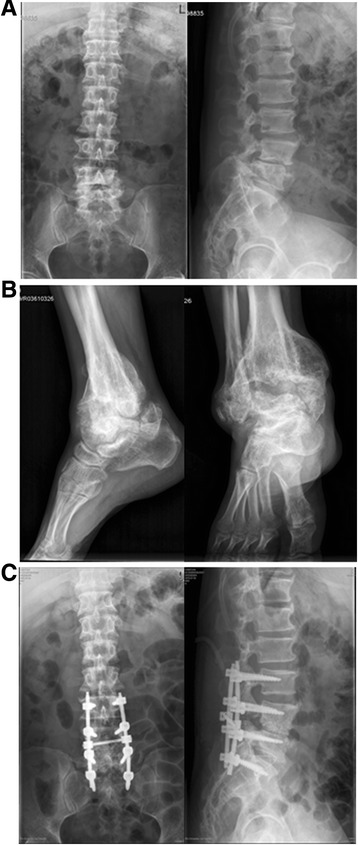
**a**. X-ray of the lumbar spine revealed narrowing of intervertebral space between L4 and L5. **b**. X-ray of left ankle showed severe destruction and disorganisation in a Charcot joint. **c**. Postoperative X-ray of lumbar spine showed well fixation and bone grafting

**Fig. 2:**
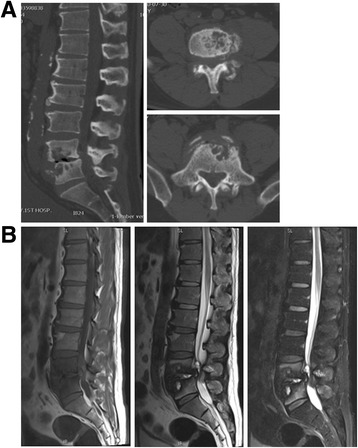
**a**. CT scan showed osteolytic lesion and new bone formation in the parts of the bodies of L4 and L5. **b**. Magnetic resonance imaging (MRI) revealed bone destruction and extraskeletal soft tissue formation posterior at the L4 vertebral body level corresponding with the stenotic area

X-ray of the lumbar spine revealed narrowing of intervertebral space between L4 and L5 (Fig. 1a). X-ray of left ankle showed severe destruction and disorganization in a Charcot joint (Fig. 1b) CT scan showed osteolytic lesion and new bone formation in the parts of the bodies of L4 and L5 (Fig. 2a). Magnetic resonance imaging (MRI) displayed that bone destruction and external skeletal soft tissue formation at the L4 vertebral body level corresponding with the stenotic area (Fig. 2b).

**Fig. 3:**
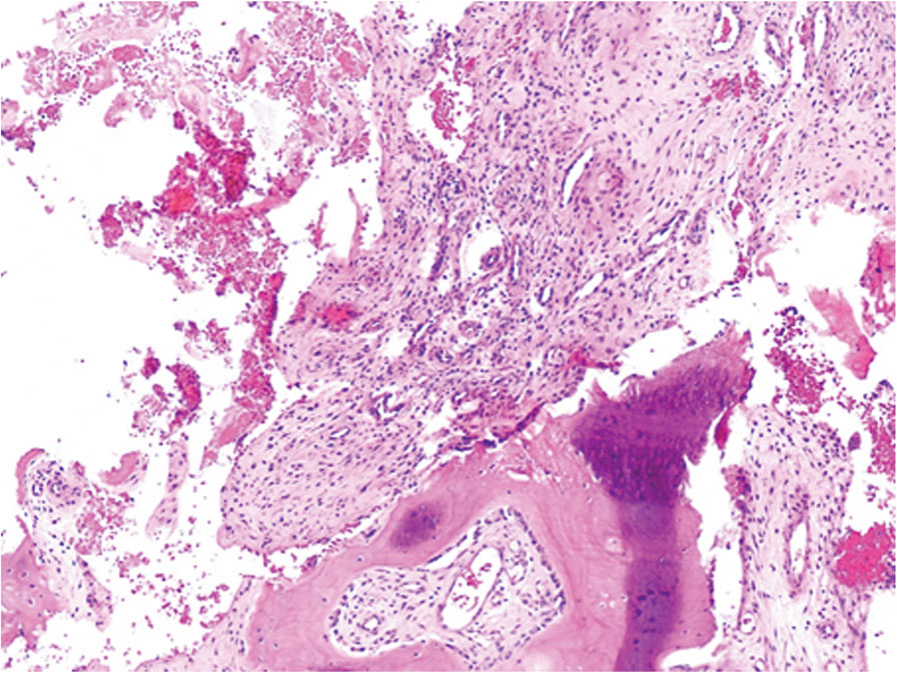
Histology showed chronic inflamed granulation tissue, degenerate bone and fibrocartilage tissue, and necrotic tissue.H&E, original magification ×20

The patient went through a posterior debridement, interbody and posterolateral allograft bone fusion with instrumentation from L3 to S1 (Fig. 1c). During operation, an organized collection was found around the dura sac and sent for histopathological examination. Histology examination showed chronic inflamed granulation tissue, degenerate bone and fibrocartilage tissue, and necrotic tissue but indistinguishable from tuberculosis (Fig. 3).

**Fig. 4:**
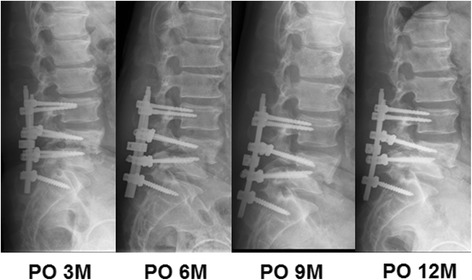
Lateral X-ray of the lumbar spine follow-up from postoperation 3 months, 6 months, 9 months and 12 months. It showed the screws were loosed and bone grafts were absorbed gradually. There is a Charcot’s arthropathy between L4 and L5. PO, postoperation

After operation, low back pain and lower extremity numbness abated immediately. The patient was diagnosed as neurosyphillis, resulting in tabetic arthropathy affecting spine and left ankle. Following the European guideline, he was treated with penicillin G benzathine 2.4 mU, intramusculary once a week, for 3 weeks after discharge [2]. During 3 months follow-up, the patient complained about low back pain and the numbness below the knees worsened again. Radiologic findings showed the screws were loosed, parts of bone grafts were absorbed and L4 and L5 were nonunion. After 6 months follow-up, the quantitative RPR reaction of serum was positive (1:4). But radiologic findings showed bone grafts were more absorbed. After 9 months and 12 months follow-up, radiologic findings showed bone grafts were absorbed totally and L4 move forward progressively (Fig. 4). Unfortunately, the patient died of acute hemorrhage caused by duodenal ulcer one and half year post-operation.

## Discussion

Syphilis is a multistage disease which is usually transmitted through sexual contact or mother-infant transmission route. The incidence of syphilis has significantly decreased by penicillin treatment during the primary and secondary stages of the disease [3, 4]. Tertiary syphilitic occurs approximately 3 to 15 years after initial infection [5]. Syphilitic involvement in the spinal column is very rare. The incidence of tertiary syphilis in the spine is not clear because this form of syphilitic complication has been documented only in occasional case reports [6–9].

The diagnosis of syphilis is facilitated by accurate and specific serological tests nowdays. But it is difficult to diagnose only with imaging and histology tests. Radiologic appearance of syphilitic spinal involvement may be misleading and resemble tuberculosis, brucellosis, degenerative osteoarthritis, or metastases [10]. Abnormal appearances on radiographs include osteophytosis, subchondral sclerosis, subluxation and soft tissue swelling. Long-term neuroarthropathy is characterized by joint dislocation [11]. It is not clear of the pathophysiology mechanism for such changes. The formation of chronic gummas is the character of gummatous syphilis. The occurrence of a gumma in bone, which is soft, tumor-like, usually causes the reactive sclerosis typical of many bony lesions. Occasionally a pure destructive lesion may be seen [12].

Up to now, T.pallidum remains sensitive to penicillin and no resistance report. However, Longer treatment duration is required for patients because the penetration of benzathine penicillin across the blood-brain barrier is poor [13]. Doxycyline or amoxicillin plus probenecid is the second line therapy option for Penicillin allergy or parenteral treatment refused [8]. However, limited data are available on efficacy of surgical treatment for tertiary syphilitic spinal lesions [7, 8, 14, 15]. Charcot’s arthropathy is one of the complicated complications and notorious to treat with or without surgical treatment [7, 11]. In our case, Charcot’s arthropathy was formed gradually even with radical debridement and interbody and posterolateral allograft bone fusion with instrumentation for destructive lesions.

## Conclusions

Our case indicates a probability of a growing number of cases presenting tertiary syphilis in the spine. It is challenging to diagnose the tertiary syphilis in the spine. Surgery is a reasonable auxiliary method to antibiotic therapy for patients who suffered with neuropathy. Charcot’s arthropathy should be considered as an operative complication.

## Abbreviations

RPR: Rapid plasma regain
TPHA: Treponema pallidum hemagglutination
